# Impaired Fertility Associated with Subclinical Hypothyroidism and Thyroid Autoimmunity: The Danish General Suburban Population Study

**DOI:** 10.1155/2015/132718

**Published:** 2015-08-17

**Authors:** Anne-Dorthe Feldthusen, Palle L. Pedersen, Jacob Larsen, Tina Toft Kristensen, Christina Ellervik, Jan Kvetny

**Affiliations:** ^1^Department of Obstetrics & Gynaecology, Naestved Hospital, Ringstedgade 61, 4700 Naestved, Denmark; ^2^The Mitochondrial Research Unit, Naestved Hospital, Ringstedgade 61, 4700 Naestved, Denmark; ^3^Department of Clinical Biochemistry, Naestved Hospital, Ringstedgade 61, 4700 Naestved, Denmark; ^4^Department of Clinical Pathology, Naestved Hospital, Ringstedgade 61, 4700 Naestved, Denmark; ^5^Department of Otorhinolaryngology-Head and Neck Surgery, Koege Hospital, Lykkebaekvej 1, 4600 Koege, Denmark; ^6^Department of Research, Nykoebing F. Hospital, 4800 Nykobing Falster, Denmark; ^7^Department of Clinical Medicine, Faculty of Health and Medical Sciences, University of Copenhagen, Denmark; ^8^Department of Internal Medicine, Naestved Hospital, Ringstedgade 61, 4700 Naestved, Denmark; ^9^Institute of Regional Health Services, University of Southern Denmark, Denmark

## Abstract

*Introduction.* The aim of this study was to estimate the significance of TSH, thyroid peroxidase antibody (TPOAb), and mild (subclinical) hypothyroidism in women from The Danish General Suburban Population Study (GESUS) on the number of children born, the number of pregnancies, and the number of spontaneous abortions. *Methods.* Retrospective cross sectional study of 11254 women participating in GESUS. Data included biochemical measurements and a self-administrated questionnaire. *Results.* 6.7% had mild (subclinical) hypothyroidism and 9.4% prevalent hypothyroidism. In women with mild hypothyroidism TPOAb was significantly elevated and age at first child was older compared to controls. TSH and TPOAb were negatively linearly associated with the number of children born and the number of pregnancies in the full cohort in age-adjusted and multiadjusted models. TSH or TPOAb was not associated with spontaneous abortions. Mild (subclinical) hypothyroidism was associated with a risk of not having children and a risk of not getting pregnant in age-adjusted and multiadjusted models. Prevalent hypothyroidism was not associated with the number of children born, the number of pregnancies, or spontaneous abortions. *Conclusion.* Impaired fertility is associated with TSH, TPOAb, and mild (subclinical) hypothyroidism in a Danish population of women.

## 1. Introduction

Through the last twenty years, the knowledge on thyroid disease during pregnancy has rapidly expanded. It is well documented that women with overt hypothyroidism during pregnancy have an increased risk of pregnancy loss and adverse pregnancy outcome [1–3], but the consequences of subclinical hypothyroidism and the significance of concomitant thyroid peroxidase antibodies (TPOAb) are debated [4–6]. Subclinical hypothyroidism is a condition in which a slightly raised thyroid stimulating hormone (TSH) signal is representing an early, mild thyroid failure [7].

The persistency of mild (subclinical) hypothyroidism may differ according to ethnicity and age; however, TSH increase with age [8] as well as the prevalence of TPOAb positivity is increasing with age [9]. Further the presence of TPOAb does seem important, as resolution to euthyroidism is reported much higher in TPOAb negative subjects compared to TPOAb positive [10]. Women with asymptomatic autoimmune thyroid disorders, who are euthyroid in early pregnancy, carry a significant risk of developing hypothyroidism progressively during gestation [11].

The aim of this study was to estimate the significance of TSH, thyroid peroxidase antibody (TPOAb), and mild (subclinical) hypothyroidism in women from The Danish General Suburban Population Study (GESUS) on the number of children born, the number of pregnancies, and the number of spontaneous abortions.

## 2. Material and Methods

### 2.1. Study Population

The Danish General Suburban Population Study (GESUS) was initiated in January 2010 and finished in October 2013. GESUS is a cross sectional study of the adult Danish suburban general population in Naestved Municipality (70 km south of Copenhagen) selected on the basis of the Danish Central Population Register Code. All individuals older than 30 years were invited. Due to financial reasons the GESUS study was designed to invite only 25% of younger women of 20–29 years age influencing the distribution of age [12].

The health examination included a comprehensive physical examination (body mass index (BMI)), biochemical tests and a self-administrated questionnaire (prevalent disease (diabetes mellitus, hypothyroidism, and hyperthyroidism), antihypertensive and cholesterol lowering medication, thyroid and antithyroid medication, contraception, smoking, income, education, employment, the number of pregnancies, the number of children born, age of first child, and the number of spontaneous abortions). The questionnaire did not include questions about induced abortion.

The GESUS study questionnaire can be viewed at http://www.p3gobservatory.org/catalogue.htm;jsessionid=ACE6E593F10B80573D64E965FA2DB3D8?measureId=38. GESUS had an overall participation rate of women of 45% (*N* = 11565). In this study we included participants of European origin (99% Danish) (*N* = 11387) and excluded participants with missing values of TSH, fT_4_, and tT_3_ (*N* = 50) and missing values of the number of pregnancies, the number of children born, and the number of spontaneous abortions (*N* = 31). Thus, we ended up with a total of 11254 women. Prevalent hypothyroidism was defined as history of hypothyroidism or intake of T_4_/T_3_ medication, and prevalent hyperthyroidism was defined as history of hyperthyroidism or intake of antithyroid medication.

### 2.2. Biochemical Variables

Thyroid hormones within the reference interval were defined as fT_4_ = 10.0–24.0 pmol/L, tT_3_ = 1.20–2.90 nmol/L, and TSH = 0.4–3.7 mU/L. Mild (subclinical) hypothyroidism was defined as TSH > 3.7 mU/L and fT_4_ and tT_3_ within the reference interval, no history of thyroid disease, and no intake of T_4_/T_3_ or antithyroid medication. Controls were defined as having 0.4 < TSH ≤ 3.7 mU/L and fT_4_ and tT_3_ within the reference interval, no history of thyroid disease, and no intake of T_4_/T_3_ or antithyroid medication [13, 14].

Thyroid peroxidase antibody (TPOAb) positivity was defined by the cut-off value of TPOAb > 60 U/mL. All biochemical tests were performed at the same laboratory at Naestved Hospital.

Measurements of TSH and of thyroid hormones-free thyroxin (fT_4_) and total triiodothyronine (tT_3_) were performed using an electrochemiluminescent immunoassay (Roche Cobas 6000, Basel, Switzerland). Reference ranges for TSH are 0.40–3.70 mU/L (CV < 7%), fT_4_ reference range 10.0–24.0 pmol/L (CV < 5%), and tT_3_ reference range 1.20–2.90 nmol/L (CV < 4%). TPOAb was measured by KRYPTOR antiTPOn (BRAHMS, Henigsdorf, Germany), with detection limit of 10 kU/L.

### 2.3. Statistics

All statistical analyses were performed using Stata version 13.0 for Windows (StataCorp, College Station, TX, USA). A value of *P* < 0.05 was considered statistically significant.

Continuous variables were compared between groups using Mann-Whitney-*U* test. Normal distribution was assessed visually using histograms and the Stata code “gladder” for the best transformation of nonnormally distributed variables. TSH and TPOAb were not normally distributed and therefore log-transformed (logTSH and logTPOAb). Categorical variables were compared using a Pearson *χ* ^2^ test. We performed the following statistical models:

1. for the full cohort of women:
  - linear regression analyses of TSH and TPOAb of children born and the number of pregnancies,
  - logistic regression for spontaneous abortion;
2. for women with prevalent hypothyroidism versus controls:
  - logistic regression analyses for ≥1 children born, ≥1 pregnancies, and spontaneous abortion;
3. for women with subclinical hypothyroidism versus controls:
  - logistic regression analyses for ≥1 children born, ≥1 pregnancies, and spontaneous abortion.

Models designed were either age-adjusted or multifactorially adjusted using age, BMI, diabetes, contraception, education, income, employment, smoking, antihypertensive medication, cholesterol lowering medication, and menopause as listed in Table 1. TPOAb was not a significant confounder in the models. There was no statistical interaction with age or menopause.

### 2.4. Ethical Considerations

The study was approved by the Regional Ethics Committee of Zealand, Denmark (Reg. number RVK SJ-177, SJ-113, and SJ-114), registered with ClinicalTrial.gov (NCT01335802), and reported to the Danish Data Protection Agency.

The study conformed to the principles of the Declaration of Helsinki. Written consent was obtained from all participants prior to participation in the GESUS study.

## 3. Results

Table 1 shows characteristics of women. In total, 758 (6.7%) had mild (subclinical) hypothyroidism, 9.4% prevalent hypothyroidism, and 4.2% prevalent hyperthyroidism. In women with mild hypothyroidism TPOAb was significantly elevated and age at first child was older compared to controls. LogTSH and logTPOAb were negatively linearly associated with the number of children born and number of pregnancies in the full cohort in age-adjusted and multiadjusted models (Table 2). logTSH or logTPOAb was not associated with spontaneous abortions.

Mild (subclinical) hypothyroidism was associated with a risk of not having children and a risk of not getting pregnant in age-adjusted and multiadjusted models. Prevalent hypothyroidism was not associated with the number of children born, the number of pregnancies, or spontaneous abortions (Table 3).

## 4. Discussion

In the present study, we showed that with higher TSH levels the less number of children born and the less number of pregnancies. Furthermore, with higher TPOAb levels the less number of children born. Coefficients for logTSH were higher than for logTPOAb; thus, the effect of TSH seems higher than for TPOAb. Mild hypothyroidism was also associated with a higher age of first child born and risk of not having children and not getting pregnant. TPOAb was not a significant confounder in the models, and there was no interaction with age.

This analysis is cross sectional and in a way retrospective for those women who are now menopausal. Also, for women who are premenopausal we may not have the life-time full number of children born, the number of pregnancies, and the number of spontaneous abortions.

The prevalence of TSH elevations increases with age [9] while the fertility declines. We only have age of the mother at the birth of her 1st child. We cannot adjust for age of the mother at the birth of her 1st child as this only applies for those who have had children; thus, those with no children have missing values in that category. We have included the information in Table 1, and it appears that there is a significant difference between those with mild hypothyroidism and controls. Furthermore, were we not able to perform analyses of pregnancy and child birth history restricted to only within few years of the TSH examination.

We were not able to detect an effect on spontaneous abortion. This may be caused by the fact that, during fertile life, early miscarriage may have gone unnoticed or been “forgotten.” Liu et al. had recently reported that women with a combination of subclinical hypothyroidism and thyroid autoimmunity were found to have the highest risk of miscarriage before 20 weeks of gestation compared to only TPOAb positivity or subclinical hypothyroidism [15]. In contrast, previous prospective studies have confirmed an association between mild hypothyroidism during pregnancy and pregnancy loss [16]. Allan et al. showed that stillbirth was significantly more frequent in women having TSH higher than 6.0 mU/L but they did not distinguish between overt and mild hypothyroidism [1]. Benhadi et al. reported that the risk of child loss was increased with higher levels of maternal TSH, whereas concentrations of maternal fT_4_ and child loss were not associated [17]. Abalovich et al. observed that the evolution of pregnancy did not depend on whether the hypothyroidism was overt or mild but depended on adequate treatment during pregnancy. If the treatment was optimized, the risk of complications was minimized [18]. These findings correspond to our results, since the odds ratios for ≥1 pregnancy and child birth versus none were not significant comparing prevalent hypothyroidism versus controls reflecting a well-treated condition. Furthermore that mild hypothyroidism is representing nonadequate treatment and mild thyroid failure was associated with a risk of not having children and a risk of not getting pregnant.

Accordingly, in our study we observed that elevated TSH above the upper reference limit in mild hypothyroidism correlated with the number of pregnancies and child births. Several studies have reported a possible association between thyroid dysfunction during pregnancy and specific adverse pregnancy outcome like preeclampsia [19, 20], placental abruption, or preterm delivery [21, 22].

It has been suggested that higher TSH and TPOAb positivity were independently associated with lower likelihood of reversion to euthyroidism [10]. In a 20-year follow-up study, an increased serum TSH level was predictive of progression to overt hypothyroidism, and the annual rate of progression to overt hypothyroidism was more than 4% in women with both raised serum TSH and antithyroid antibodies [23]. Glinoer et al. showed a trend towards slightly higher serum TSH levels in women with thyroid autoimmunity in the first trimester of pregnancy compared to women without thyroid autoimmunity and that women with asymptomatic autoimmune thyroid disease who were euthyroid in early pregnancy carried a significant risk of developing hypothyroidism progressively during gestation, despite a marked reduction in antibody titers [11].

This retrospective cross sectional study showed that among women without any history of thyroid disease or antihyperthyroid/antihypothyroid medication 6.7% had mild (subclinical) hypothyroidism. The prevalence of mild hypothyroidism was comparable to previous studies [24] like the Colorado study in which 8.5% had subclinical hypothyroidism increasing with age [25] explained by the chosen value of TSH cut-off defining mild hypothyroidism.

The presence of mild hypothyroidism influenced the level of fT_4_ within the reference range as fT_4_ was decreased in women with mild hypothyroidism. This could be explained by an increased intracellular deiodination of T_4_ to T_3_ in women with mild hypothyroidism to avoid decreased levels of active intracellular thyroid hormone concentrations. A major problem is to determine separate effects of TPOAb positivity and mild hypothyroidism. We are not able to determine the status during pregnancy as the present study is retrospective, but it seems unlikely that TPOAb would have an effect directly on metabolism and peripheral thyroid hormone regulated cellular function. However, we cannot exclude the possibility that the women presenting with mild hypothyroidism have had a previous normalized TSH level.

This population study was performed in Naestved Municipality eastern Denmark representing a mild iodine deficiency [26]. In Denmark, the iodine intake was stable at a low level for many years and in 2000, the mandatory iodine fortification of bread salt and household salt began. Follow-up studies reported an increase in prevalence of hypothyroidism [27] and TPOAb positivity especially among younger women [28].

The present study is limited by the fact that all clinical observations are self-reported questionnaire data. Furthermore, we did not have any information about the numbers of induced abortions which could influence the data of child births although it is rarely recommended to induce an abortion only due to high levels of TSH during first trimester of pregnancy.

In addition, individuals were classified based on a single blood test; thus, we cannot distinguish between transient and permanent TSH elevation; however, Somwaru et al. showed that subclinical hypothyroidism was persistent in 56% after 4-year follow-up [10]. Finally, due to financial reasons the GESUS study was designed to invite only 25% of younger women aged 20–30 years old influencing the distribution of age.

The strength of our study was the high number of participants with blood samples of TSH, fT_4_, tT_3_, and TPOAb in 11254 women. Furthermore, for the analyses of mild (subclinical) hypothyroidism we excluded women with any self-reported thyroid disease or use of T_4_/T_3_ or antithyroid medication and only compared euthyroid and mild hypothyroid women.

## 5. Conclusion

Taken together, we observed that with higher TSH levels the less number of children born and the less number of pregnancies. Furthermore, with higher TPOAb levels the less number of children born. Mild hypothyroidism was also associated with a higher age of first child born and risk of not having children and not getting pregnant. In conclusion, we observed that impaired fertility is associated with TSH, TPOAb, and mild (subclinical) hypothyroidism in a Danish population of women.

## Figures and Tables

**Table 1:** Characteristics of women in The Danish General Suburban Population Study (GESUS).

	All	Mild (subclinical) hypothyroidism		
		No	yes	*P*-value
*N* (%)	11254 (100)	8770	758	
Age	56.3 (45.5–66.2)	55.4 (44.8–65.6)	55.6 (44.9–65.9)	0.43
Menopause, yes (%)	7129 (63.4)	5405 (61.6)	463 (61.1)	0.77
TSH, mU/L	1.8 (1.2–2.6)	1.7 (1.2–2.3)	4.6 (4.1–5.5)	<0.001
Total T3, nmol/L	1.6 (1.5–1.9)	1.7 (1.5–1.9)	1.6 (1.5–1.8)	0.38
Free T4, pmol/L	15 (14–17)	15 (14–17)	14 (13–15)	<0.001
TPOAb, U/mL	13 (20–32)	19 (12–27)	28 (15–699)	<0.001
Body mass index (BMI), kg/m²	25.3 (22.6–28.8)	25.2 (22.5–28.7)	25.5 (22.8–29.2)	0.06
Smoker, yes (%)	1899 (16.9)	1527 (17.5)	66 (8.7)	<0.001
Prevalent hypothyroidism, yes (%)	922 (9.4)	NA	NA	
Prevalent hyperthyroidism, yes (%)	391 (4.2)	NA	NA	
Diabetes mellitus, yes (%)	576 (5.1)	392 (4.5)	40 (5.3)	0.31
Antihypertensive medication, yes (%)	2457 (21.8)	1816 (20.7)	140 (18.5)	0.14
Cholesterol lowering medication, yes (%)	1510 (8.5)	1096 (12.5)	91 (12.0)	0.69
Contraception, yes (%)	953 (8.5)	772 (8.8)	66 (8.7)	0.93
Income below EUR 60,000 €	4744 (43.7)	3588 (42.4)	292 (40.1)	0.25
Unemployment, yes (%)	6284 (55.8)	3690 (42.1)	318 (42.0)	0.95
Education, no (%)	1713 (15.2)	1291 (14.7)	95 (12.5)	0.57
Age at 1st child born	25 (22–28)	25 (22–28)	25 (22–29)	0.02
No children born, *N* (%)	1171 (10.5)	897 (10.3)	98 (13.0)	0.02
No pregnancies, *N* (%)	952 (8.5)	733 (8.4)	77 (10.2)	0.09
Spontaneous abortion, yes (%)	2261 (21)	1747 (20.8)	149 (20.6)	0.87

For continuous variables: median (interquartile range).

For SCH, *P* value: Chi-square for categorical comparisons and ranksum or Kruskal-Wallis test for continuous comparisons.

**Table 2:** Association between TSH (mU/L) and TPOAb (U/mL) levels and the number of children born, the number of pregnancies, and spontaneous abortion.

	Age-adjusted		Multiadjusted^*∗*^	
	Coefficient (95% CI)	*P* value	Coefficient (95% CI)	*P* value
	Number of children born			
log⁡TSH	−0.046 (−0.068–(−0.023))	<0.001	−0.050 (−0.072–(−0.0272))	<0.001
log⁡TPOAb	−0.020 (−0.037–(−0.003))	0.02	−0.021 (−0.038–(−0.004))	0.02

	Number of pregnancies			
log⁡TSH	−0.081 (−0.132–(−0.030))	0.002	−0.085 (−0.136–(−0.033))	0.001
log⁡TPOAb	−0.019 (−0.055–0.017)	0.31	−0.021 (−0.058–0.016)	0.27

	Spontaneous abortion (yes versus no)			
log⁡TSH	0.96 (0.91–1.01)	0.12	0.95 (0.89–1.01)	0.08
log⁡TPOAb	1.00 (0.95–1.04)	0.86	1.00 (0.96–1.04)	0.94

^*∗*^Multiadjusted model: age, menopause, BMI, smoking, diabetes, antihypertensive medication, cholesterol lowering medication, contraception, income, unemployment, and education.

Correlation between TSH and TPOAb and number of children born and number of pregnancies are measured was by linear regression.

Association between TSH and TPOAb and spontaneous abortion (yes vs. no) was by logistic regression.

**Table 3:** Table of odds ratios for ≥1 children born, ≥1 pregnancy, and spontaneous abortion in patients with prevalent hypothyroidism versus controls and in patients with mild (subclinical) hypothyroidism versus controls.

	Prevalent hypothyroidism				Mild (subclinical) hypothyroidism			
	Odds ratio (95% CI)	*P* value	Odds ratio (95% CI)	*P*-value	Odds ratio (95% CI)	*P*-value	Odds ratio (95% CI)	*P*-value
	Age-adjusted		Multiadjusted^*∗*^		Age-adjusted		Multiadjusted^*∗*^	
≥1 children born versus none	0.84 (0.67–1.06)	0.14	0.87 (0.69–1.10)	0.25	0.75 (0.60–0.94)	0.01	0.71 (0.56–0.90)	0.004
≥1 pregnancy versus none	0.83 (0.65–1.07)	0.15	0.85 (0.66–1.1)	0.22	0.79 (0.61–1.01)	0.06	0.75 (0.58–0.97)	0.03
Spontaneous abortion (yes versus no)	1.05 (0.88–1.24)	0.6	1.02 (0.89–1.22)	0.78	0.99 (0.82–1.19)	0.87	0.96 (0.79–1.17)	0.69

^*∗*^Multiadjusted model: age, menopause, BMI, smoking, diabetes, antihypertensive medication, cholesterol lowering medication, contraception, income, unemployment, and education.

## Abbreviations

BMI: Body mass index
fT_4_: Free thyroxine
GESUS: Danish General Suburban Population Study
SCH: Mild (subclinical) hypothyroidism
TPOAb: Thyroid peroxidase antibody
TSH: Thyroid stimulating hormone
tT_3_: Total triiodothyronine.
