# Corrigendum to “Impaired Fertility Associated with Subclinical Hypothyroidism and Thyroid Autoimmunity: The Danish General Suburban Population Study”

**DOI:** 10.1155/2017/9864034

**Published:** 2017-11-05

**Authors:** Anne-Dorthe Feldthusen, Palle L. Pedersen, Jacob Larsen, Tina Toft Kristensen, Christina Ellervik, Jan Kvetny

**Affiliations:** ^1^Department of Obstetrics & Gynaecology, Naestved Hospital, Ringstedgade 61, 4700 Naestved, Denmark; ^2^The Mitochondrial Research Unit, Naestved Hospital, Ringstedgade 61, 4700 Naestved, Denmark; ^3^Department of Clinical Biochemistry, Naestved Hospital, Ringstedgade 61, 4700 Naestved, Denmark; ^4^Department of Clinical Pathology, Naestved Hospital, Ringstedgade 61, 4700 Naestved, Denmark; ^5^Department of Otorhinolaryngology-Head and Neck Surgery, Koege Hospital, Lykkebaekvej 1, 4600 Koege, Denmark; ^6^Department of Research, Nykoebing F. Hospital, 4800 Nykobing Falster, Denmark; ^7^Department of Clinical Medicine, Faculty of Health and Medical Sciences, University of Copenhagen, Denmark; ^8^Department of Internal Medicine, Naestved Hospital, Ringstedgade 61, 4700 Naestved, Denmark; ^9^Institute of Regional Health Services, University of Southern Denmark, Denmark

In the article titled “Impaired Fertility Associated with Subclinical Hypothyroidism and Thyroid Autoimmunity: The Danish General Suburban Population Study” [1], there were errors in Materials and Methods and Results described as follows.

In Section “2.1. Study Population,” the sentence “All individuals older than 30 years were invited” should be changed to “All individuals older than or equal to 30 years were invited.” In addition, the sentence “In this study we included participants of European origin (99% Danish) (*N* = 11387) and excluded participants with missing values of TSH, fT4, and tT3 (*N* = 50) and missing values of the number of pregnancies, the number of children born, and the number of spontaneous abortions (*N* = 31)” should be changed to “In this study we included participants of European origin (99% Danish) (*N* = 11387) and excluded participants with missing values of TSH, fT4, and tT3 (*N* = 50) and missing values of the number of pregnancies, the number of children born, and the number of spontaneous abortions (*N* = 83).”

In “Results,” the sentence “Table  1 shows characteristics of women. In total, 758 (6.7%) had mild (subclinical) hypothyroidism, 9.4% prevalent hypothyroidism, and 4.2% prevalent hyperthyroidism” should be changed to “Table 1 shows characteristics of women. In total, 758 (6.7%) had mild (subclinical) hypothyroidism, 8.2% prevalent hypothyroidism, 3.5% prevalent hyperthyroidism, and 4.2% prevalent hyperthyroidism.” Therefore, Table  1 should be corrected as follows.

## Figures and Tables

**Table 1:** Characteristics of women in The Danish General Suburban Population Study (GESUS).

	All	Mild (subclinical) hypothyroidism			
		No	Yes	*P* value	
*N* (%)	11254 (100)	8770	758		
Age	56.3 (45.5–66.2)	55.4 (44.8–65.6)	55.6 (44.9–65.9)	0.43	
Menopause, yes (%)	7129 (63.4)	5405 (61.6)	463 (61.1)	0.77	
TSH, mU/L	1.8 (1.2–2.6)	1.7 (1.2–2.3)	4.6 (4.1–5.5)	<0.001	
Total T3, nmol/L	1.6 (1.5–1.9)	1.7 (1.5–1.9)	1.6 (1.5–1.8)	0.38	
Free T4, pmol/L	15 (14–17)	15 (14–17)	14 (13–15)	<0.001	
TPOAb, U/mL	*20 (13–32)*	19 (12–27)	*28 (15–698)*	<0.001	
Body mass index (BMI), kg/m^2^	25.3 (22.6–28.8)	25.2 (22.5–28.7)	25.5 (22.8–29.2)	0.06	
Smoker, yes (%)	1899 (16.9)	1527 (17.5)	66 (8.7)	<0.001	
Prevalent hypothyroidism, yes (%)	*922 (8.2)*	NA	NA		Corrected percentage: 8.2%
Prevalent hyperthyroidism, yes (%)	*391 (3.5)*	NA	NA		Corrected percentage: 3.5%
Diabetes mellitus, yes (%)	576 (5.1)	392 (4.5)	40 (5.3)	0.31	
Antihypertensive medication, yes (%)	2457 (21.8)	1816 (20.7)	140 (18.5)	0.14	
Cholesterol lowering medication, yes (%)	*1510 (13.4)*	1096 (12.5)	91 (12.0)	0.69	Corrected percentage: 13.4%
Contraception, yes (%)	953 (8.5)	772 (8.8)	66 (8.7)	0.93	
Income below EUR 60,000 €	4744 (43.7)	3588 (42.4)	292 (40.1)	0.25	
Unemployment, yes (%)	*4970 (44.2)*	3690 (42.1)	318 (42.0)	0.95	Corrected *N* = 4970 (44.2%)
*No* ^*∗*^ * education, N (%)*	1713 (15.2)	1291 (14.7)	95 (12.5)	0.57	
Age at 1st child born	25 (22–28)	25 (22–28)	25 (22–29)	0.02	
No^*∗*^ children born, *N* (%)	*1173 (10.5)*	897 (10.3)	98 (13.0)	0.02	
No^*∗*^ pregnancies, *N* (%)	952 (8.5)	733 (8.4)	77 (10.2)	0.09	
Spontaneous abortion, yes (%)	2261 (21.0)	1747 (20.8)	149 (20.6)	0.87	

For continuous variables: median (interquartile range); for SCH, *P* value: chi-square for categorical comparisons and ranksum or Kruskal-Wallis test for continuous comparisons; *for some of the variables, there were missing values. The percentage is therefore of the total with information on the variable and not the total N* = 11254*; *^*∗*^*No*  *= never*.
